# Biopsy-proven autoimmune myocarditis in HIV-associated dilated cardiomyopathy

**DOI:** 10.1186/s12879-014-0729-3

**Published:** 2014-12-31

**Authors:** Andrea Frustaci, Nicola Petrosillo, Marco Francone, Romina Verardo, Giuseppe Ippolito, Cristina Chimenti

**Affiliations:** Biocardiology Laboratory, National Institute for Infectious Diseases “Lazzaro Spallanzani”, IRCCS, Via Portuense, 292, Rome, 00149 Italy; Radiological Sciences Department, “La Sapienza” University, Rome, Italy

**Keywords:** HIV (Human immunodeficiency virus), Biopsy (Heart), Myocarditis, Heart failure, Recovery of function

## Abstract

**Background:**

Dilated cardiomyopathy occurring in HIV-infected patients raises both diagnostic and therapeutic challenging problems. Indeed myocardial involvement in HIV infection has been variously attributed to several causes, including viral, toxic, nutritional and autoimmune, but no specific treatment capable to substantially improve patients’ prognosis has been recognized so far.

**Case Presentation:**

Hereby we describe the case of an autoimmune myocarditis manifesting with heart failure in a3 9-year-old man with HIV infection.

Left ventricular endomyocardial biopsy showed a lymphocytic myocarditis characterized by over-expression of HLA-DR and negative polymerase chain reaction for cardiotropic viruses. Steroid treatment was followed by recovery of cardiac dimension and function.

**Conclusion:**

Presence of auto-reactive myocarditis should be considered in patients with HIV-associated dilated cardiomyopathy. Its recognition by endomyocardial biopsy followed by steroid administration may result in a complete resolution of cardiac disease.

**Electronic supplementary material:**

The online version of this article (doi:10.1186/s12879-014-0729-3) contains supplementary material, which is available to authorized users.

## Background

Occurrence of dilated cardiomyopathy (DCM) in patients with HIV infection raises particular challenging and investigational problems; indeed it is believed that the intrinsic as well as the contamination risks of invasive procedures like endomyocardial biopsy, outweigh the clinical benefit it may derive from the knowledge of the histological and molecular cardiac substrate. However, although myocardial involvement in HIV infection has been variously attributed to HIV itself [1],[2], opportunistic and viral infections [3], drug related cardiac toxicity [4], nutritional deficiencies [5] and autoimmune response to viral infection [6], no specific treatment capable to substantially improve patients’ prognosis has been recognized so far. As a consequence, HIV-related DCM is usually managed with a supportive, anti-failing heart therapy registering very often a grim outcome. In the following study we report a chronic severe cardiac dilatation and dysfunction in a HIV-infected patient, caused by an auto-reactive myocarditis, the recognition of which through endomyocardial biopsy has led to a complete recovery after steroid administration.

## Case presentation

**Figure 1:**
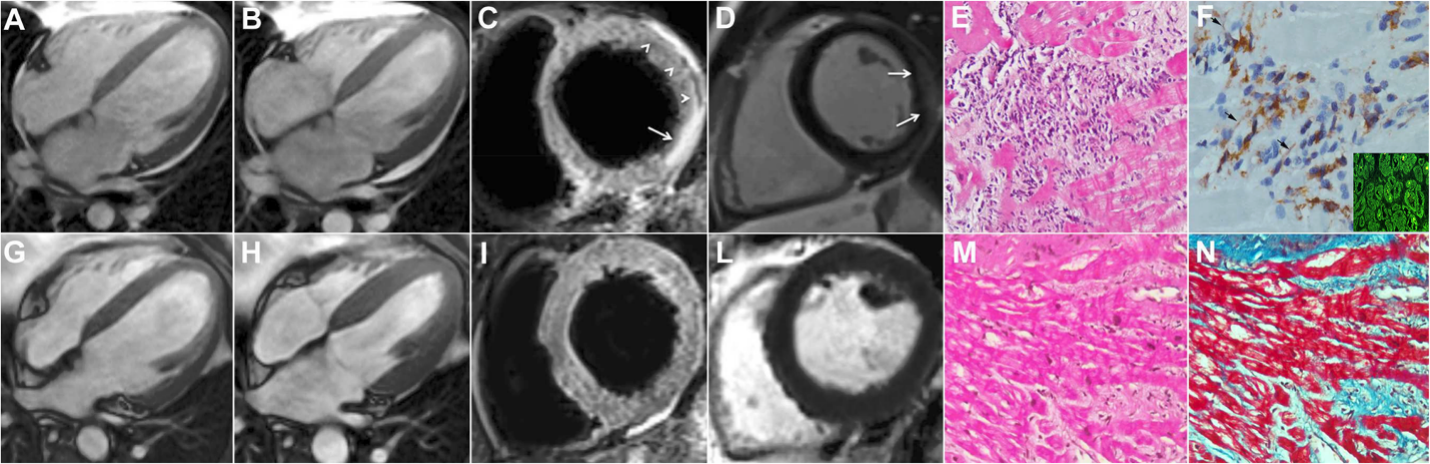
**Cardiac magnetic resonance and left ventricular endomyocardial biopsy before (upper panels) and after recovery from autoimmune myocarditis (lower panels) in HIV- infected patient.** Long axis images (panel **A** = diastole, panel **B** = systole) show a severe dilated and dysfunctional left ventricle recovering (ejection fraction from 20 to 45%) after 4 months of steroid treatment (panel **G** = diastole, panel **H** = systole). T2 short-tau inversion recovery images (T2-STIR) in mid ventricular short axis show subepicardial edematous imbibition of the infero-lateral segment of the left ventricular myocardium (panel **C**, arrows), and thickening of pericardial layers with minimal amount of effusion (panel **C**, arrowheads) corresponding to late gadolinium enhancement (LGE) with the same distribution (panel **D**, arrows). At 4-month follow up T2w-STIR e LGE images (panel **I** and **L**) show complete regression of tissue edema and late enhancement. Severe lymphocytic myocarditis (panel **E**, H&E, 200x) with overexpression of HLA-DR on cardiomyocyte membrane (arrow in panel **F**, immunoperoxidase, 400x) and positivity of cardiac serum to antiheart autoantibodies (panel **F** insert) resulted in healed myocarditis with disappearance of inflammatory infiltrates (panel **M**, H&E, 200x) and interstitial and focal replacement fibrosis (panel **N**, Masson’s trichrome, 200x).

A 39-year-old HIV infected caucasian man was admitted because of progressive heart failure beginning 9 months earlier after an episode of upper respiratory tract infection. His clinical history was marked by a serious compromise of lymphocyte count (CD4+ lymphocytes 95/cmm) and high blood viral load (1.3 × 10^6^ copies/ml) at the time of HIV infection recognition at the age of 29 years. He experienced several infectious events including gastroenteritis, pneumonia and toxoplasma encephalitis before the introduction of anti- retroviral therapy seven years before. Since then he was under antiretroviral treatment (ritonavir, darunavir and emtricitabine/tenofovir) registering a recovery of lymphocyte count (CD4+ up to 500/cmm) and an undetectable viral load in the peripheral blood. The time course of heart failure symptoms and laboratory/instrumental changes from antiretroviral therapy was about seven years. His heart failure was characterized by progressive dyspnea, absence of fever and chest pain but mild and constant increase of troponin T (between 0.1 and 0.8 μg/L, normal values < 0.014). The electrocardiogram documented a sinus tachycardia (125 bts/min) with low QRS voltages while 2D-echocardiogram showed normal valvular pattern but a progressive bi-ventricular dilatation (right and left ventricular end diastolic diameter was 36 and 68 mm respectively) and dysfunction (RV and LV ejection fraction 35 and 25%) despite an optimized dose of carvedilol (50 mg bid), digoxin (0.125 mg/daily), enalapril (20 mg bid) and furosemide (up to 250 mg/daily). Cardiac magnetic resonance showed a left ventricular (LV) end-diastolic volume of 231.6 ml (Figure 1A), a LV end-systolic volume of 184.2 ml (Figure 1B) and an ejection fraction of 20% with remarkable edema (Figure 1C) and delayed gadolinium enhancement of the lateral LV free wall (Figure 1D), with minimal pericardial effusion.

Due to the presence of chronic, severe (NYHA class IV), drug-resistant heart failure the patient underwent an invasive cardiac study including coronary with LV angiography and a LV endomyocardial biopsy with histology and polymerase chain reaction for a large panel (HIV, Hepatitis C virus, Human Herpes Virus 6, Human Herpes Virus 8, Herpes Simples virus ½, Epstein Barr virus, Adenovirus, Cytomegolovirus, Enterovirus, Influenza A/B, Parvovirus B19) of cardiotropic viruses on 6 samples. Coronary network was normal while at histology a severe active lymphocytic myocarditis was observed characterized by intense cell necrosis (Figure 1E), marked expression of HLA-DR on cardiomyocyte membrane (Figure 1F) and absence of detectable viral genomes, including HCV. Serology for anti-DNA, anti-cardiolipin and ANCA antibodies was negative while it was positive for ANA (++/+++). Cardiac specific autoantibodies were searched for by means of indirect immunofluorescence as previously described [7] and showed a strong positivity in heart muscle while were negative in skeletal muscle (Figure 1F insert). Supportive treatment was, then, implemented by high dose steroid administration (methylprednisolone 1 gr IV/daily for 3 days followed by prednisolone 40 mg IV/bid for 1 week and then prednisone 1 mg/kg/daily orally) with rapid improvement of general and cardiac conditions: in one week sinus tachycardia reduced from 125 to 78 bts/min, gallop rhythm was no more appreciable and LVEF rose to 28%; after 4 months the patient reached a NYHA class I regaining a normal social life, electrocardiogram registered a heart rate of 62 bts/min with increase of QRS voltages and improvement of ventricular repolarization while a new 2D-echocardiogram showed remarkable reduction of LV volumes with nearly normal LVEF. At control cardiac magnetic resonance, LV end-diastolic volume reduced to 140.6 ml (from 231.6) (Figure 1G), LV end-systolic volume declined to 77.7 ml (from 184.2) (Figure 1H) and LVEF increased to 45% (from 20%) while myocardial edema (Figure 1I) as well as delayed enhancement (Figure 1L) and pericardial effusion were no more appreciable.

Control histology showed a resolution of the inflammatory process (Figure 1M) with interstitial and replacement fibrosis (Figure 1N).

## Discussion and conclusions

The underlying myocardial damage in DCM has been attributed to various causes and mechanisms [1]-[6], and its assesment requires to submit the patient to invasive cardiac studies including coronary angiography and endomyocardial biopsy. The latter procedure is sometimes not performed because of the risk of unfavorable cardiac events and of potential HIV exposure for healthcare workers. On the other hand, use of non-invasive exams, including cardiac magnetic resonance, is of limited informative value: indeed, the possible observation of edema, hyperemia and delayed gadolinium enhancement, suggesting the presence of myocarditis, does not provide any causal information useful to establish a specific treatment [8]. On the opposite, histological and molecular study of endomyocardial biopsies can confirm the presence of myocarditis, define through immunohistochemistry the pathologic type of inflammatory infiltrate (lymphocytic, eosinophilic, granulomatous, giant cell), identify by means of polymerase chain reaction the offending infectious agent and then indicate an appropriate therapeutic regimen. In particular new promising antiviral agents as beta-interferon [9] are emerging to contrast viral myocarditis, immunoglobulins [10] and immunoadsorption [11] are a current option for virus-positive and virus-negative respectively inflammatory cardiomyopathy, while the recent prospective, randomized TIMIC study [7] has given encouraging results (cardiac improvement in 88% of treated patients) for the immunosuppressive treatment of virus- negative myocarditis. The present report suggests that auto-reactive myocarditis, suggested by the positivity to anti-heart autoantibodies [12] is, perhaps, an underestimated cause of DCM in HIV-infected patients and that its recognition, through endomyocardial biopsy may lead to a prompt steroid treatment with the recovery of cardiac dimensions and function even in subjects in a terminal phase of heart failure. The mechanism involved likely includes the recovery of immune-competence of lymphocytes by the administration of antiretroviral agents and the lymphocytes’ reaction toward segregated myocyte antigens possibly shared by viral structural components.

Finally HIV itself is supposed to modify lymphocyte gene expression [13], leading eventually to the induction of an autoimmune process. The auto-reactive nature of inflammatory damage has been documented in our report by negative polymerase chain reaction for a large panel of cardiotropic viruses, the prominent HLA-DR expression in the sarcolemmal membrane of myocardiocytes and the extraordinary response to steroid administration of a chronically failing heart unresponsive to an optimized supportive therapy. This observation encourages the invasive investigation of HIV-associated DCM and the histological/molecular analysis of endomyocardial tissue samples. In conclusion presence of auto-reactive myocarditis should be considered in patients with HIV-associated DCM. Its recognition by endomyocardial biopsy followed by steroid administration may result in a complete resolution of cardiac disease.

## Consent

Written informed was obtained from the patient for publication of this case report and any accompanying images. A copy of the written consent is available for review by the Editor of this journal.

## Authors’ original submitted files for images

Below are the links to the authors’ original submitted files for images. Authors’ original file for figure 1

## References

[1] Hajjar LA, Calderaro D, Yu PC, Guiliano I, de Oliviera LEM, Barbaro G, Caramelli B (2005). Cardiovascular manifestations in patients infected with the human immunodeficiency virus. Arq Bras Cardiol.

[2] Sani MU (2008). Myocardial disease in human immunodeficiency virus (HIV) infection: a review. Wien Klin Wochenschr.

[3] Calabarese LH, Proffitt MR, Yen-Lieberman B, Hobbs RE, Ratliff NB (1989). Congestive cardiomyopathy and illness related to the acquired immunodeficiency syndrome (AIDS) associated with isolation of retrovirus from myocardium. Ann Intern Med.

[4] Fantoni M, Autore C, Del Borgo C (2001). Drugs and cardiotoxicity in HIV and AIDS. Ann NY Acad Sci.

[5] Hoffman M, Lipshultz SE, Miller TL, Miller TL, Gorbach S (1999). Malnutrition and cardiac abnormalities in the HIV-infected patients. Nutritional aspects of HIV infection.

[6] Herskowitz A, Willoughby SB, Vlahov D, Baughman KL, Ansari AA (1995). Dilated heart muscle disease associated with HIV infection. Eur Heart J.

[7] Frustaci A, Chimenti C, Calabrese F, Pieroni M, Thiene G, Maseri A (2003). Immunosuppressive therapy for active lymphocytic myocarditis: virological and immunologic profile of responders versus nonresponders. Circulation.

[8] Caforio AL, Pankuweit S, Arbustini E, Basso C, Gimeno-Blanes J, Felix SB, Fu M, Heliö T, Heymans S, Jahns R, Klingel K, Linhart A, Maisch B, McKenna W, Mogensen J, Pinto YM, Ristic A, Schultheiss HP, Seggewiss H, Tavazzi L, Thiene G, Yilmaz A, Charron P, Elliott PM, European Society of Cardiology Working Group on Myocardial and Pericardial Diseases (2013). Current state of knowledge on aetiology, diagnosis, management, and therapy of myocarditis: a position statement of the European Society of Cardiology Working Group on Myocardial and Pericardial Diseases. Eur Heart J.

[9] Kühl U, Lassner D, von Schlippenbach J, Poller W, Schultheiss HP (2012). Interferon-Beta improves survival in enterovirus-associated cardiomyopathy. J Am Coll Cardiol.

[10] Drucker NA, Colan SD, Lewis AB, Beiser AS, Wessel DL, Takahashi M, Baker AL, Perez-Atayde AR, Newburger JW (1994). Gamma-globulin treatment of acute myocarditis in the pediatric population. Circulation.

[11] Schultheiss HP, Kühl U, Cooper LT (2011). The management of myocarditis. Eur Heart J.

[12] Currie PF, Goldman JH, Caforio AL, Jacob AJ, Baig MK, Brettle RP, Boon NA, McKenna WJ (1998). Evidence of cardiac autoimmunity in HIV related heart muscle disease. Heart.

[13] Via CS, Morse HC, Shearer GM (1990). Altered immunoregulation and autoimmune aspects of HIV infection: relevant murine models. Immunol Today.

