# Supplementary figures and images for: Biopsy-proven autoimmune myocarditis in HIV-associated dilated cardiomyopathy
Source: BMC Infect Dis. 2014 Dec 31;14:729. doi: 10.1186/s12879-014-0729-3 (PMC4325940; doi:10.1186/s12879-014-0729-3)

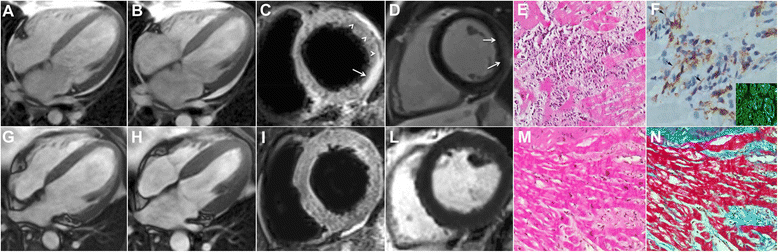

Supplement: Supplementary file 1 — Authors’ original file for figure 1 [file 12879_2014_729_MOESM1_ESM.gif]
